# Contemporary adjuvant polymethyl methacrylate cementation optimally limits recurrence in primary giant cell tumor of bone patients compared to bone grafting: a systematic review and meta-analysis

**DOI:** 10.1186/1477-7819-11-156

**Published:** 2013-07-16

**Authors:** Dongqing Zuo, Longpo Zheng, Wei Sun, Dong Fu, Yingqi Hua, Zhengdong Cai

**Affiliations:** 1 Department of Musculoskeletal Oncology, Shanghai Tenth People’s Hospital, Tongji University School of Medicine, Shanghai 200072, China

**Keywords:** Giant cell tumors, Recurrence, Polymethyl methacrylate, Cementation, Bone grafting

## Abstract

**Background:**

Reports of recurrence following restructuring of primary giant cell tumor (GCT) defects using polymethyl methacrylate (PMMA) bone cementation or allogeneic bone graft with and without adjuvants for intralesional curettage vary widely. Systematic review and meta-analysis were conducted to investigate efficacy of PMMA bone cementation and allogeneic bone grafting following intralesional curettage for GCT.

**Methods:**

Medline, EMBASE, Google Scholar, and Cochrane databases were searched for studies reporting GCT of bone treatment with PMMA cementation and/or bone grafting with or without adjuvant therapy following intralesional curettage of primary GCTs. Pooled risk ratios and 95% confidence intervals (CIs) for local recurrence risks were calculated by fixed-effects methods.

**Results:**

Of 1,690 relevant titles, 6 eligible studies (1,293 patients) spanning March 2008 to December 2011 were identified in published data. Treatment outcomes of PMMA-only (*n* = 374), bone graft-only (*n* = 436), PMMA with or without adjuvant (PMMA + adjuvant; *n* = 594), and bone graft filling with or without adjuvant (bone graft + adjuvant; *n* = 699) were compared. Bone graft-only patients exhibited higher recurrence rates than PMMA-treated patients (RR 2.09, 95% CI (1.64, 2.66), Overall effect: Z = 6.00; *P* <0.001), and bone graft + adjuvant patients exhibited higher recurrence rates than PMMA + adjuvant patients (RR 1.66, 95% CI (1.21, 2.28), Overall effect: Z = 3.15, *P* = 0.002).

**Conclusions:**

Local recurrence was minimal in PMMA cementation patients, suggesting that PMMA is preferable for routine clinical restructuring in eligible GCT patients. Relationships between tumor characteristics, other modern adjuvants, and recurrence require further exploration.

## Background

Giant cell tumors (GCTs) of bone are primary bone tumors of mesenchymal origin that commonly present as localized osteolytic lesions in the epiphysiometaphyseal region of long bones, though these tumors also occur with relatively lower frequency in other bone monostotic processes [1]. GCTs account for up to 20% of all primary skeletal neoplasms [1] and 5% of all adult primary bone tumors [2]. Recurrence reports in patient subpopulations vary, ranging from 0% to 65% [2].

For benign to locally aggressive GCT tumors, recurrence is most common in local tissues due to narrow surgical margins [3]; however, 3.5% of GCT patients develop remote benign or metastatic lesions [4-6], with malignancy variably reported in ≤30% of cases [7]. Diverse presentations, diagnostic methods, and treatment methodologies may contribute to discrepancies between reports [7,8]. Since the relatively recent decline of affected limb amputation as a preferred treatment, contemporary clinical course and proper treatment selection have become more challenging [9]. Though modern curettage with local adjuvant or *en bloc* excision with prosthetic reconstruction are widely accepted treatment strategies for GCT of bone, consistently reported to reduce recurrence compared to wide excision [10-17], there is no consensus for optimal surgical curettage methodology, including fillers and adjuvants, to limit recurrence.

In routine intralesional curettage for GCTs of bone, adjuvants, such as the thermal adjuvant polymethyl methacrylate (PMMA) and chemical adjuvant phenol, have been recommended to reduce local recurrence following intralesional surgery, resulting in disease-free survival rates as high as 85% [2]. PMMA cementation treatment after curettage immediately stabilizes the affected limb and releases heat during polymerization that may kill remaining tumor cells [18,19], achieving recurrence rates ranging from 12 to 65.2% in various reports [13,20]. For lesions near the articulating surface, subchondral allogeneic bone grafting is also a widely accepted alternative for filling voids during intralesional curettage either with or without additional adjuvants, with recurrence rates comparable to PMMA treatment [2].

Despite the prevalence of studies concerning GCT and its recurrence, little conclusive data and no widely accepted consensus for optimal surgical management and adjuvant selection for GCT of bone are available. The current study investigates the efficacy of PMMA bone cementation and allogeneic bone grafting following intralesional curettage for surgical management of GCT of bone through a systematic review and meta-analysis, thus providing evidence for clinical treatment selection.

## Methods

### Study design

A systematic literature search was performed to identify cohort studies assessing efficacy and recurrence of primary GCT following intralesional curettage treatment with only PMMA bone cementation (PMMA-only), only allogeneic bone grafting (bone graft-only), PMMA bone cementation with or without adjuvant (PPMA + adjuvant), and allogeneic bone grafting with or without adjuvant (bone graft + adjuvant). Results were systematically analyzed to determine the relationship between treatment methods and recurrence rates in PMMA-treated and bone graft-treated patients.

### Inclusion and exclusion criteria

Studies were included that reported information pertaining to efficacy and recurrence of GCT of bone following treatment with PMMA bone cementation or allogeneic bone grafting with or without other adjuvants. All included studies (1) contained patients who underwent intralesional curettage for treatment of pathologically verified primary GCTs; (2) reported void filling with either PMMA or allogeneic bone graft; (3) reported recurrence rates following intralesional curettage with ≥2 treatment groups for efficacy assessments; and (4) reported a ≥3 year follow-up period. All included studies were also (5) published or previously translated into in the English language. Studies that (1) did not include a retrospective control group or (2) contained patient cohorts sized ≤30 patients for any group were excluded.

### Database search terms

Electronic searches were performed using the electronic databases provided by Google Scholar (1966 to September 2012), Medline (1966 to September 2012), EMBASE (1974 to September 2012), and the Cochrane Controlled Trial Register (Cochrane library 2012). Two independent researchers conducted literature searches using the search keywords ‘bone cement’, ‘PMMA’, ‘polymethyl methacrylate’, ‘bone graft’, ‘giant cell tumor of bone’, and ‘recurrence’ with various combinations of the operators ‘AND’, ‘NOT’, and ‘OR’.

### Quality assessment

Eligible studies were evaluated for inclusion by two independent reviewers (Zuo and Hua), and the level of agreement between reviewers was recorded. Inclusion of resultant titles was determined by screening of manual titles and abstracts, followed by full-text screening by the same reviewers. The quality of each study was assessed using the Methodological Index for Nonrandomized Studies (MINORs) scoring system [21] and the Newcastle Ottawa Quality Assessment Scale (NOQAS). These scales were used to allocate a maximum of nine points for quality of selection, comparability, exposure, and outcome of study participants. In the event of incomplete data, authors of potentially eligible studies were contacted to obtain relevant unpublished data.

### Outcome measurement

Local recurrence was the primary endpoint for analysis. Recurrence was defined as radiological and pathological evidence of local disease recurrence necessitating further surgical intervention.

### Statistical analysis

All data were analyzed using RevMan v.5.1 software (Cochrane Collaboration, Copenhagen, Denmark). Risk ratio (RR) and 95% confidence intervals (CIs) were reported. Heterogeneity among studies was assessed using Cochrane’s Q test with a *P* value equal to 0.1. An *I*-squared (variability) statistic is the percentage of total variation across studies due to heterogeneity. A random effects model was used for heterogeneous data; otherwise, a fixed effect model was used. Meta-analysis of pooled risk ratios was performed. *P* values less than 0.05 were considered statistically significant (*P* <0.05).

## Result

### Literature search

**Figure 1:**
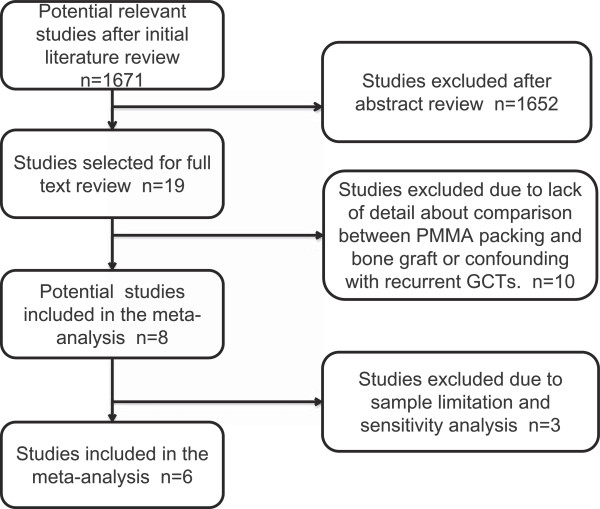
Flow diagram detailing study inclusion.

Initial electronic database searches yielded 1,690 relevant titles, of which 1,671 were excluded due to failure to meet the inclusion criteria. The remaining 19 articles were subjected to full-text review, resulting in exclusion of 8 additional articles due to failure to meet the inclusion criteria, most commonly due to inappropriate comparison methods. Additionally, two articles [10,17] were excluded due to insufficient primary GCT data. Notably, in both cases, original data was not able to be obtained from the corresponding authors. Furthermore, although three studies [22-24] included in the meta-analysis conducted by Liu *et al*. [25] met the inclusion criteria, these studies were excluded due to the relatively small number of included cases (35 PMMA-only and 20 bone graft-only). Study inclusion is detailed in Figure 1. Systematic review and meta-analysis were conducted using the remaining six included studies [2,11,12,14,15,20].

### Study characteristics and quality assessment

In these 6 included retrospective cohort studies, data were reported for a total of 1,293 patients with primary GCTs of bone treated with either intralesional curettage or other resection methodologies. Median follow-up times for each study ranged from 60 to 108 months. Publication dates ranged from March 2008 to December 2011. Quality assessments revealed average NOQAS scores from the two reviewers of 7.4 and 7.0, indicating that all six included studies were of moderate quality.

PMMA treatment was administered to 594 patients, and the remaining 699 patients underwent no PMMA treatment. Among the total 1,293 patients, 436 patients were treated only with intralesional curettage and bone graft, and 373 patients were treated only with intralesional curettage and PMMA cementation. Patient demographics, follow-up, and lesion characteristics are detailed in Table 1.

**Table 1:** The details of the six articles included in the current review and a meta-analysis

**Study**	**Publication date**	**Follow-up**	**Patient no.**	**Surgical treatment groups**	**Patient**	**Recurrence rate**	**Recurrence ( *n*)**
	**(year)**	**(months)**					
Becker *et al.*[20]	2008	63	384	Total cohort			
			78/384	wide resection		2.00%	1
			103/384	curettage (bone graft)		49.00%	56
			102/384	curettage + PMMA		22.00%	23
			74/384	curettage + PMMA + phenol		27.00%	20
			27/384	curettage + toxic(bone graft)		15.00%	4
Errani *et al.*[11]	2010	91	349	Total cohort			
			149/349	wide resection		12.00%	18
			136/349	Curettage + burr + phenol		17.60%	24
			64/349	Curettage + burr + phenol + PMMA		12.50%	8
Kivioja *et al.*[15]	2008	60 months	294	Total cohort			
			92/294	wide resection		12.00%	11
			47/294	curettage		51.00%	24
			147/294	curettage + PMMA		22.00%	32
Klenke *et al.*[2]	2010	108 months	118	Total cohort			
			UN	wide resection		5.00%	unknown
			22/118	curettage + burr		32.00%	7
			32/118	curettage + burr + phenol		34.00%	11
			41/118	curettage + burr + phenol + PMMA		15.00%	6
Gaston *et al.*[12]	2011	76.5 months	330	Total cohort			
			246/330	curettage		28.70%	67
			84/330	curettage + PMMA		14.30%	12
Jamshidi *et al.*[14]	2008	74 months	168	Total cohort			
			40/168	curettage + bone		42.50%	17
			40/168	curettage + cement		30.00%	12
			46/168	curettage + burr + bone		21.70%	10
			42/168	curettage + burr + cement		16.70%	7
Harness *et al*. [22]	2004	72 months	31				
			5/31	curettage + bone		40.00%	2
			26/31	curettage + PMMA		42.00%	11
Ozalp *et al.*[23]	2006	80 months	8	Total cohort			
			6/8	curettage + bone		50.00%	3
			2/8	curettage + PMMA		50.00%	1
Sheth *et al.*[24]	1995	108 months	16	Total cohort			
			9/16	curettage + bone		33.00%	3
			7/16	curettage + PMMA		28.50%	2

### Heterogeneity of studies

Variability (*I*-squared) in the results of the four studies used to compare PMMA-only and bone graft-only patients demonstrated a true difference in treatment effect of 0%, indicating no heterogeneity. In all six studies used to compare PPMA + adjuvant and bone graft + adjuvant patients, *I*-squared values were 56%, indicating relatively high heterogeneity. Thus, a random effects model was employed to adjust for heterogeneity prior to comparison of PPMA + adjuvant and bone graft + adjuvant patients.

### Local recurrence

**Figure 2:**
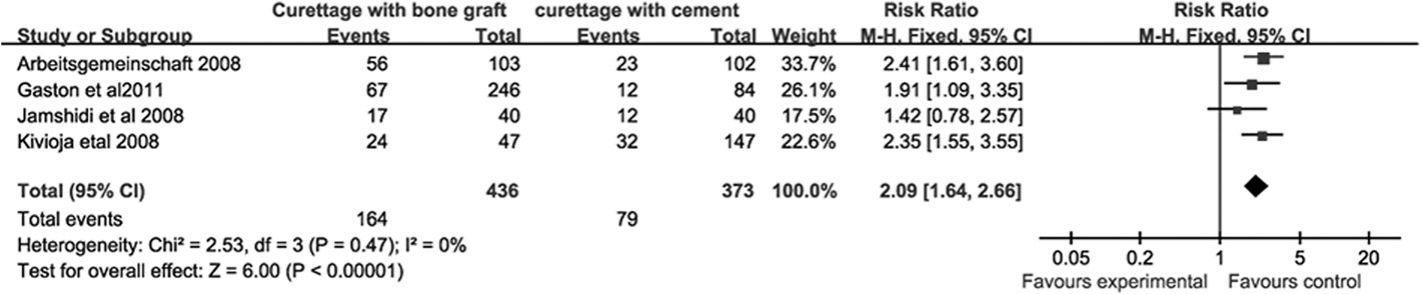
**Forest plot for recurrence comparison between patients with intralesional curettage followed by polymethyl methacrylate (PMMA) cementation alone (PMMA-only) or bone grafting (bone graft-only).**

**Figure 3:**
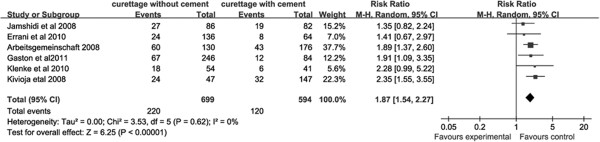
Forest plot for recurrence comparison between patients with and without treatment with polymethyl methacrylate (PMMA) cementation following intralesional curettage.

Complete data for recurrence was available in all included studies, and all six studies were included in analysis of local recurrence. Local recurrence rates differed significantly between PMMA-only patients and bone graft-only patients, with bone graft-only patients exhibiting significantly higher recurrence rates than PMMA-only patients (RR = 2.09, 95% CI (1.64, 2.66), Overall effect: Z = 6.00; *P* <0.001; Figure 2). Local recurrence rates also differed between PPMA + adjuvant patients and bone graft + adjuvant patients. Bone graft + adjuvant patients also demonstrated higher levels of recurrence compared to PPMA + adjuvant patients (RR = 1.87, 95% CI (1.55, 3.55), Test for overall effect: Z = 6.25; *P* <0.001; Figure 3).

## Discussion and conclusions

In order to provide an accurate and contemporary analysis of GCT of bone treatments, the current meta-analysis was conducted of six methodologically sound studies, encompassing 1,293 GCT patients. Included patients were treated with intralesional curettage followed by PMMA cementation or allogeneic bone grafting, with results clearly indicating that PMMA cementation more optimally reduces recurrence risk in these patients. Despite extensive recent investigations of the relationships between adjuvant use and treatment efficacy, recurrence, and GCT of bone aggression, no consensus for proper treatment has been widely accepted. Thus, the current study provides novel and compelling evidence supporting preferential use of PMMA cementation in contemporary clinical intralesional curettage for GCT of bone.

No wide consensus has been reached for preferential use of PMMA or bone graft treatments in GCT of bone, with conflicting recent evidence attributable primarily to methodological discrepancies between studies. In a multicenter retrospective study of sarcoma in 294 Scandinavian patients at 13 centers followed for a median of 5 years, Kivioja *et al*. [15] reported that PMMA cementation significantly reduced local recurrence rates following intralesional curettage, supporting widespread preferential use of PMMA treatments. Similar results were reported in a 384-patient, multicenter study conducted by Becker *et al*. [20], wherein 22% of GCT patients treated with PMMA and 49% of GCT patients treated with bone graft exhibited local recurrence within 63 months, evidence that further supports the use of PMMA treatments. Conversely, Liu *et al.*[25] conducted a systematic review and meta-analysis of 80 intralesional curettage patients with GCT of the distal radius, reporting that PMMA cementation was not an effective adjuvant compared with bone graft, with PMMA treatments exhibiting both higher rates of local recurrence and metastatic events. These discrepancies may be due to methodological differences as well as the fast-paced changes in routine surgical practices over the last several decades, indicating that contemporary evaluation of these methods is required. Thus, a consensus pertaining to treatment standards for GCT has not yet been reached, though the current study provides powerful, contemporary evidence to support preferential PMMA adjuvant use in modern clinical settings.

Without the use of adjuvants, reoccurrence rates following intralesional curettage as high as 35 to 49% have been reported [2,20]. Increases in available adjuvant types and numbers in recent years have, however, further complicated the treatment selection process for GCT patients, though few studies have conducted systematic comparisons of the cytotoxic characteristics of specific adjuvant formulations [26]. As a result, the vast selection of clinically-approved adjuvants may be overwhelming for many clinicians, resulting in default use of sub-optimal older methods due to subjective comfort levels rather than clinical viability [26]. Several reports have reported dramatic decreases in recurrence rates in patients treated with PMMA, PMMA with phenol, or phenol with other chemical adjuvants (22%, 27%, and 15% recurrence, respectively) [3,16,27,28]. Niu *et al*. [16] reported that extended curettage with combined high speed burring, bone graft, and PMMA cementation resulted in an extremely low recurrence rate of 3.3%, though numerous other studies refute these findings and provide conflicting results [14,17,25,29,30]. Algawahmed *et al.*[29] conducted a systematic review and meta-analysis of 387 GCT patients, demonstrating that meticulous surgical techniques, including high-speed burring, are paramount to reducing GCT recurrence rates. Furthermore, these findings and those of the current study further indicated that local adjuvants, such as PMMA, were not associated with recurrence. Similarly, a meta-analysis of 139 GCT patients with symptoms in the distal radius conducted by Liu *et al*. [25] suggested that tumor recurrence in patients treated with PMMA did not differ significantly from that observed in patients treated with bone grafting.

In the current meta-analysis, only four articles were used to assess PMMA-only and bone-graft only treatments [12,14,15,20], while six articles were used to assess PMMA and bone grafts applied either with or without other adjuvants following intralesional curettage [2,11,12,14,15,20]. The current findings used pooled data to reveal significant differences in both comparison groups, with primary GCTs patients treated with PMMA cementation following intralesional curettage exhibiting relatively lower recurrence rates than patients treated with bone grafts following intralesional curettage. It has been hypothesized that the additional heat produced by the exothermic reaction of *in situ* PMMA polymerization may contribute to removal of remaining tumor cells in the curetted cavity of GCT lesions [15], thereby reducing recurrence. Similarly, it has been suggested that abnormal mitosis, increased mitotic rate (>10 mm^2^), and permeation of vascular channels may increase tumor aggressiveness and recurrence rates [7], thereby making adjuvant treatment with PMMA less useful due to the larger distribution of tumor cells in local tissues.

While these compounding factors may, in part, explain the controversial findings reported by different studies, further clinical, radiologic, and histologic investigation will be required to assess the potential relationships between adjuvant treatments and the myriad of contemporary compounding factors. Based on both previous findings and current experience, it is hypothetically possible that tumor volume may also influence recurrence of primary GCTs. Logically, both PMMA cementation and bone graft treatments are likely to be less effective following intralesional curettage in cases of very small tumors, which are more difficult to excise and fill [31]. Because this raises the likelihood that some tumor cells will elude the hypothetical thermal effects of PMMA, such adjuvant treatment is likely to be less effective in GCTs with very small volumes. This volume-effect hypothesis, however, will require further clinical exploration.

Despite the relatively large number of patients studied, the limitations of meta-analyses containing only a small number of studies must be considered. Due to the relative scarcity of both the disease and relevant, reputable randomized control trials, other meta-analyses in this field have also been conducted using very small groups of articles, including the studies by Algawahmed *et al.*[29] and Liu *et al.*[25]. The largest number of included patients were contributed by the report of Becker *et al.*[20], consisting of 384 patients. These patients included 256 primary GCT cases and 128 recurrent GCT cases. Notably, failure to distinguish between primary and recurrent GCT cases in the Becker *et al*. study is a potentially confounding factor in the current study. The difference between local recurrence rates between primary and recurrent tumors was found to be negligible in this study (21.9% versus 23.4%), and the other two studies that included both primary and recurrent GCT cases [10,17] were excluded due to significant differences between primary local recurrence and overall recurrent rates (33.5% versus 20% and 35% versus 18%, respectively). Furthermore, adjuvants applied along with PMMA and bone graft treatments following curettage were not distinguished, and future study will be required to assess whether different adjuvant combinations may alter outcomes.

It is important to note that when only local recurrence outcomes are assessed, as in the current study, the effects of potentially important parameters may be overlooked, such as metastatic rate, abnormal mitoses, and related major complication rates. Szalay *et al.*[32] and Gaston *et al.*[12] suggested that joint cartilaginous degeneration may occur following PMMA cementation in joint arthroplasty. Similarly, Gaston *et al.*[12] evaluated the rate of joint replacement for patients with GCTs, revealing that patients treated with PMMA evidenced significantly higher rates of joint replacement. While metastasis in GCTs patients is rare [33,34], up to 3% of metastatic GCT occurrences are found in pulmonary tissues [11,35]. Notably, only three studies [2,11,12] included in the current analysis provided discrepant results relating to metastasis and function. While this data is obviously insufficient to conduct a current analysis of these functional results, this provides an interesting topic for further study.

The current study of pooled data from 1,293 primary GCTs cases in six studies in contemporary medical literature, revealed that PMMA cementation following intralesional curettage produced lower recurrence rates compared to recurrence rates in patients treated with bone grafting. Recent advancements in GCT of bone treatment methodologies have produced numerous compounding factors that must be considered in order to achieve better outcomes in these patients, making modern adjuvant formulations and surgical methodologies critical aspects of treatment selection. Thus, this study provides critically needed systematic, evidence-based evidence indicating that PMMA is a superior treatment for the majority of GCT of bone cases treated in routine clinical practice. To better evaluate new and improved adjuvant treatments, however, additional multicenter studies with sufficient and comprehensive data regarding tumor characteristics will be required.

## Abbreviations

GCT: Giant cell tumor; MINORs: Methodological Index for Nonrandomized Studies; NOQAS: Newcastle Ottawa Quality Assessment Scale; RR: Risk ratio.

## Competing interests

The authors declare that they have no competing interests.

## Authors’ contributions

ZD, ZL carried out the studies, participated in collecting data, and drafted the manuscript. SW, FD performed the statistical analysis and participated in its design. HY, CZ helped to draft the manuscript. All authors read and approved the final manuscript.
